# Unusual Antioxidant Properties of 26S Proteasome Isolated from Cold-Adapted Organisms

**DOI:** 10.3390/ijms18081605

**Published:** 2017-07-25

**Authors:** Marta Gogliettino, Ennio Cocca, Carmela Fusco, Bruna Agrillo, Alessia Riccio, Marco Balestrieri, Angelo Facchiano, Antonio Pepe, Gianna Palmieri

**Affiliations:** 1 Institute of Biosciences and BioResources, National Research Council (CNR-IBBR), Via Pietro Castellino 111, 80131 Napoli, Italy; marta.gogliettino@ibbr.cnr.it (M.G.); carmela.fusco@ibbr.cnr.it (C.F.); bruna.agrillo@ibbr.cnr.it (B.A.); alessia.riccio@ibbr.cnr.it (A.R.); marco.balestrieri@ibbr.cnr.it (M.B.); antonio.pepe@ibbr.cnr.it (A.P.); gianna.palmieri@ibbr.cnr.it (G.P.); 2 Institute of Food Sciences, National Research Council (CNR-ISA), Via Roma 64, 83100 Avellino, Italy; angelo.facchiano@isa.cnr.it

**Keywords:** oxidative stress, 26S proteasome, oxidized protein degradation, cold-adaptation, antioxidant functions, Antarctic fish

## Abstract

The oxidative challenge represents an important factor affecting the adaptive strategies in Antarctic fish, but their impact on the protein degradation machinery still remains unclear. The previous analysis of the first 26S proteasome from the Antarctic red-blooded fish *Trematomus bernacchii*, evidenced improved antioxidant functions necessary to counteract the environmental pro-oxidant conditions. The purpose of this work was to carry out a study on 26S proteasomes from the temperate red-blooded *Dicenthrarcus labrax* and the icefish *Chionodraco hamatus* in comparison with the isoform already described from *T. bernacchii*, to better elucidate the cold-adapted physiological functions of this complex. Therefore, the 26S isoforms were isolated and the complementary DNAs (cDNAs) codifying the catalytic subunits were cloned. The biochemical characterization of Antarctic 26S proteasomes revealed their significantly higher structural stability and resistance to H_2_O_2_ with respect to that of the temperate counterpart, as also suggested by a comparative modeling analysis of the catalytic subunits. Moreover, in contrast to that observed in *T. bernacchii*, the 26S systems from *C. hamatus* and *D. labrax* were incapable to hydrolyze oxidized proteins in a ubiquitin-independent manner. Therefore, the ‘uncommon’ properties displayed by the Antarctic 26S proteasomes can mirror the impact exercised by evolutionary pressure in response to richly oxygenated environments.

## 1. Introduction

Protein oxidation is a natural consequence of aerobic metabolism. Oxygen, although essential for most living organisms, is described as a Janus-faced molecule, as it is a powerful oxidant capable of ‘wreaking havoc’ on biological macromolecules when it forms reactive oxygen species (ROS), which are generated by cellular metabolism and/or environmental sources [1]. These radical compounds are unstable and highly reactive and can interact with various cellular components, causing considerable damage to vital molecules, such as proteins [2,3]. Indeed, an irreversible oxidation of proteins can induce structural and/or functional alterations or even switch off their catalytic properties [4]. Hence, because life has become aerobic and inefficient removal of these compounds results in cytotoxicity, living cells have developed many protective systems to counteract the ROS-induced oxidative damage and maintain redox homeostasis [5]. In mammals, the ubiquitin–proteasome system represents one of the major proteolytic systems responsible for most intracellular protein degradation [6]. The proteasome is a multicatalytic complex that exists in two major forms—20S and 26S [7]. It is well established that the majority of oxidatively-modified proteins are degraded in an adenosine triphosphate (ATP)- and ubiquitin-independent pathway by the catalytic 20S “core”, while the 26S proteasome plays a role in the ubiquitin-dependent protein degradation processes [2,5,8,9]. In accordance with this, the 20S proteasome is much more resistant to oxidative stress than the 26S isoform, which loses activity and is easily inactivated even at low concentration of oxidants such as hydrogen peroxide [10,11]. Therefore, 20S proteasome is considered one of the most important machinery of the antioxidant defense systems in living cells [12,13]. Oxidative stress phenomena in polar ectotherms have recently become of greater interest in climate adaptation research, due to higher oxygen solubility in cold seawater and body fluids of these organisms, which elevates rates of cellular ROS production [14,15]. Hence, the life at the permanently cold- and oxygen-rich waters is associated with increased tissue oxygenation, which is responsible for higher oxidative stress levels in polar aquatic systems, as compared to temperate counterparts [14]. This condition has created the need to adapt the antioxidant defenses that have allowed such organisms to readily accommodate the burst of ROS generation [14,16]. In this context, Antarctic fish represent unparalleled model organisms for studying the oxidative stress phenomena, due to their physiological and biochemical adaptations arisen during millions of years of evolution [16,17].

Recently, we provided the first evidence on the role of the 26S proteasome in the antioxidant defense systems in the Antarctic notothenioid *Trematomus bernacchii*, suggesting that the cold-adaptation may have had a greater effect on the 26S antioxidant capacity, making it more stable and especially able to degrade oxidized proteins via a ubiquitin-independent pathway [18], differently from the isoform purified from mammalian cells [5,9,10,11,19]. In this context, to further investigate whether the ‘atypical’ antioxidant capacities displayed by the *T. bernacchii* 26S proteasome were the result of the cold-adaptation or an intrinsic feature of the piscine isoforms [18], our study was extended by conducting a comparative analysis with the isoforms isolated from the white-blooded *Chionodraco hamatus* and the warm red-blooded *Dicenthrarcus labrax*. One of the most curious traits of *C. hamatus*, an Antarctic fish belonging to the family *Channichthyidae* (icefish), has the lack of erythrocytes and the oxygen-binding protein hemoglobin (Hb), evidenced by its milky-white blood [1]. As iron-centered proteins, Hb can promote the formation of ROS that may damage macromolecules. Therefore, icefish have developed outstanding compensatory re-adaptations to accommodate the lack of hemic pigment and enhance oxygen delivery, including extensive circulatory, vascular and cardiac adjustments [20,21]. In addition, the loss of Hb reduces the production of ROS, the levels of oxidized proteins and the energetic costs associated with replacing oxidatively damaged proteins [1]. Herein, we purified and characterized 26S proteasomes from *C. hamatus* and *D. labrax* blood cells and compared their properties with those of the already described red-blooded *T. bernacchii* isoform [18]. This analysis provided a further piece of the cold-adaptation puzzle, supporting the idea that the ‘uncommon’ 26S antioxidant properties displayed from the Antarctic fish may represent a response needed to potentiate antioxidant defenses and counteract the higher levels of oxidative stress to which these organisms are exposed.

## 2. Results and Discussion

### 2.1. Isolation and Purification of 26S Proteasome from Dicenthrarcus labrax and Chionodraco hamatus Erythrocytes

**Figure 1:**
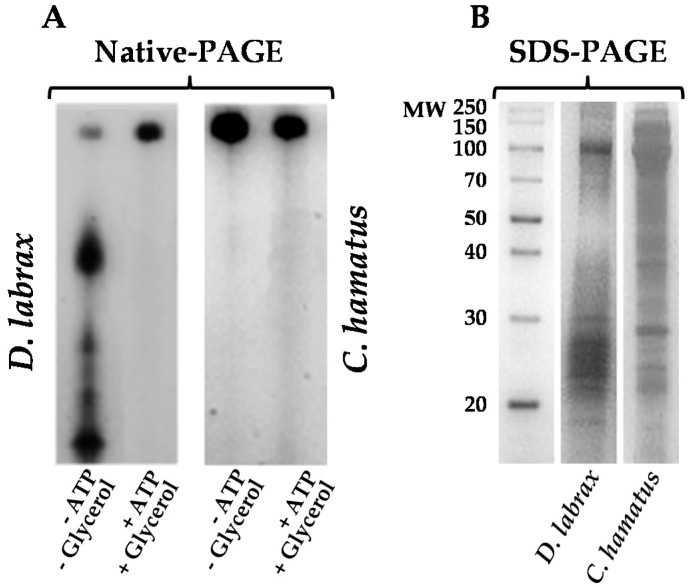
Native-polyacrylamide gel electrophoresis (Native-PAGE) (**A**) and sodium dodecyl sulfate–polyacrylamide gel electrophoresis (SDS-PAGE) (**B**) analyses of purified *Dicenthrarcus labrax* and *Chionodraco hamatus* 26S proteasomes followed by Coomassie blue staining. The results obtained with or without adenosine triphosphate (ATP; 1 mM) and glycerol (10%) during the purification procedures are shown for both fish. The results are representative of three independent experiments on three different protein preparations.

To gain insight into the antioxidant functions of 26S proteasome in organisms living under cold-induced oxidative stressful conditions, a comparative study of this complex purified from the icefish *C. hamatus* and the temperate specie *D. labrax*, was carried out. Firstly, we optimized a purification strategy (Tables S1 and S2) to obtain the intact 26S proteasome from blood cells of both fish. Proteasome enrichment was followed by monitoring the chymotrypsin (CT)-like activity using LLVY (*N*-succinyl-Leu-Leu-Val-Tyr-7-amido-4-methylcoumarin) as substrate. Notably, all buffers used to isolate *D. labrax* 26S proteasome (Figure 1) included ATP (1 mM) and glycerol (10%), in order to prevent its dissociation into 19S and 20S particles, as reported for mammalian counterparts [22,23]. Instead, as already reported for *T. bernacchii* proteasome [18], glycerol and ATP were not essential to purify to homogeneity the *C. hamatus* 26S isoform and to preserve its integrity as shown in Native-polyacrylamide gel electrophoresis (Native-PAGE) gel (Figure 1A), suggesting a noticeable structural stability of the holoenzyme respect to that of the temperate isoform. In addition, sodium dodecyl sulfate–polyacrylamide gel electrophoresis (SDS-PAGE) analysis reported in Figure 1B, clearly displayed the wide range of molecular masses (20–150 kDa) for both the purified 26S proteasomes, as typically observed in the eukaryal counterparts [7,24].

**Figure 2:**
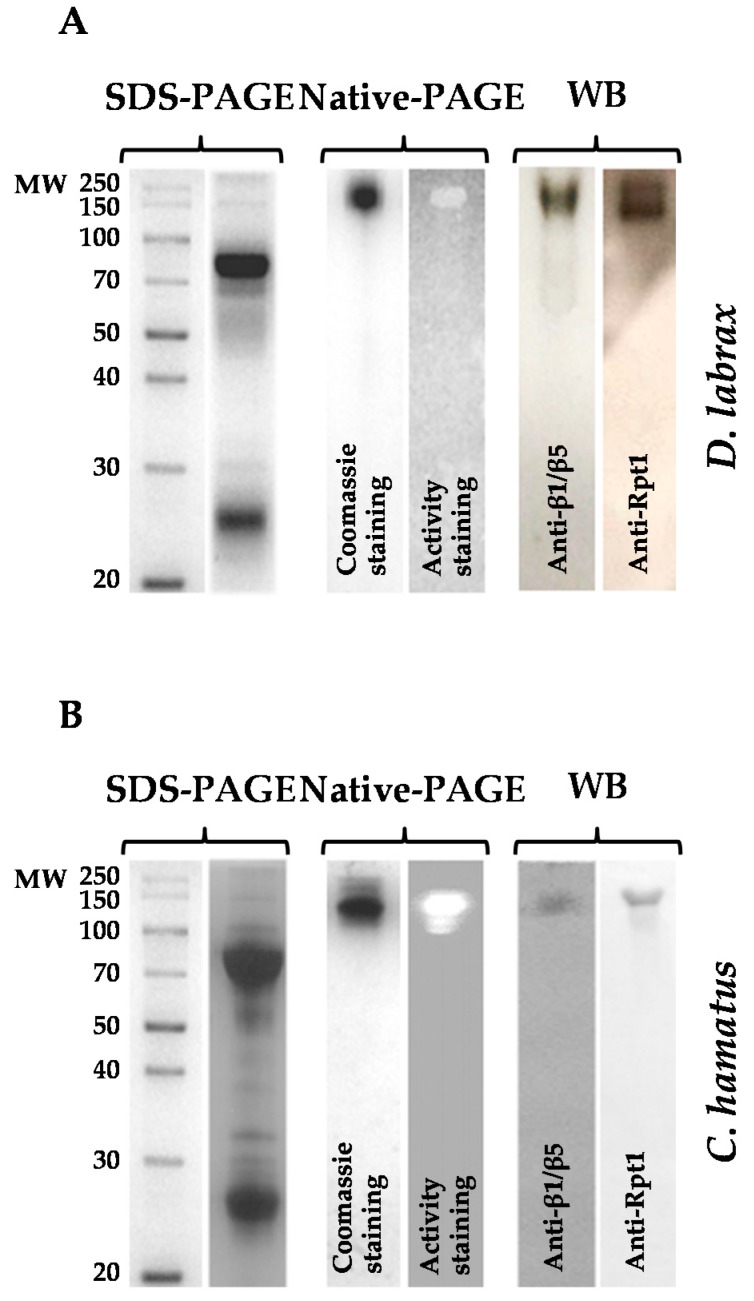
SDS-PAGE, Native-PAGE and Western blot analyses of 26S proteasomes purified from *D. labrax* (**A**) and *C. hamatus* (**B**). Native-PAGE analysis of 26S proteasomes was developed either by in-gel detection of chymotrypsin (CT)-like activity (β5 subunit), using the fluorogenic substrate LLVY (*N*-succinyl-Leu-Leu-Val-Tyr-7-amido-4-methylcoumarin), that by Coomassie blue staining. Native-PAGE of 26S proteasomes was immunoblotted against Rpt1t (subunit of 19S) or β1/β5 (subunits of 20S) antibodies. The results are representative of three independent experiments on three different protein preparations.

**Figure 3:**
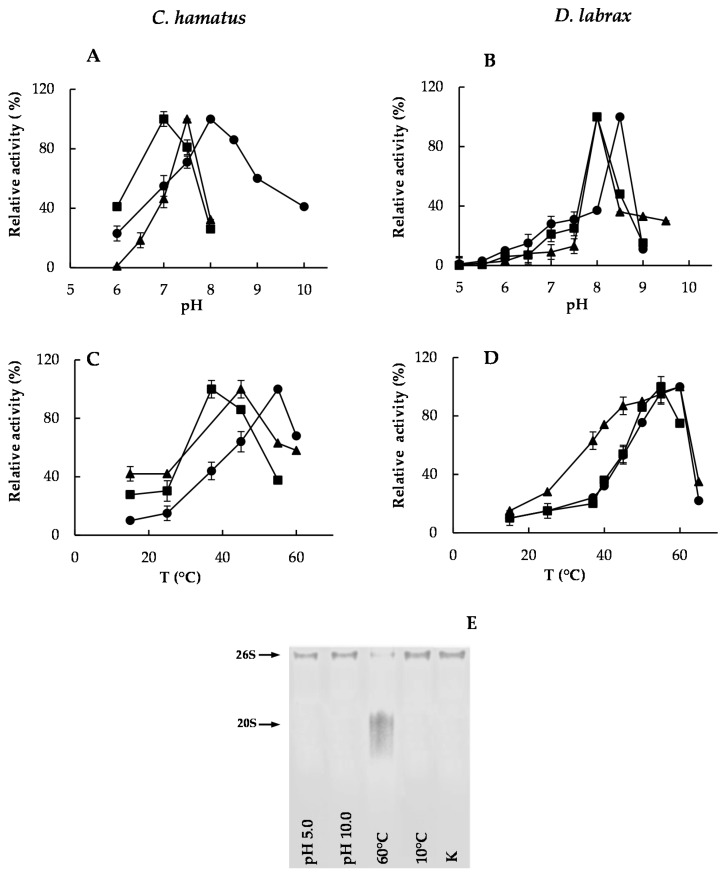
Molecular properties of purified *C. hamatus* or *D. labrax* 26S proteasomes. (**A**,**B**) pH and (**C**,**D**) temperature effects on CT-like (circle), post-glutamate peptide hydrolase (PGPH)-like (square) and trypsin (T)-like (triangle) activities of 26S proteasomes. Relative activities are expressed as percentage of the corresponding maximal activities. All experiments were performed in triplicate on three different protein preparations. Three blank measurements (with no enzyme) at each pH and temperature value were performed. (**E**) Coomassie blue stained Native-PAGE of 26S proteasome from *C. hamatus* after pre-incubation for 5 min at extreme pH and temperature values. 26S proteasome pre-incubated for 5 min at pH 7.5 and 37 °C (K) was used as control. Data are expressed as means ± standard deviations. Standard deviation values lower than 5% are not shown.

The identity of the isolated 26S complexes was confirmed by different analytical procedures: (1) gel filtration chromatography, which allowed to estimate a molecular mass of 2000 kDa, accordingly to the eukaryal isoforms [24,25,26]; (2) Native-PAGE, followed by in-gel detection of CT-like activity and Coomassie blue staining, evidencing a single band corresponding to the 26S isoform (Figure 2A,B); (3) immunoblot analyses in native conditions, using specific antibodies both against β1/β5 (belonging to 20S) or Rpt1 (belonging to 19S) subunits. In addition, in-gel CT-like activity (β5 subunit) assay of 26S proteasomes was also performed in the presence of SDS (Figure S1) to further confirm the absence of free 20S core particles in the purified samples, using 26S proteasomes from *T. bernacchii* and human as positive controls.

### 2.2. pH and Temperature Effects on Chymotrypsin-, Trypsin- and PGPH-Like Activities of Piscine 26S Proteasomes

A comparative analysis of the pH and temperature effects on the peptide-hydrolyzing activities: CT-like; caspase (post-glutamate peptide hydrolase (PGPH))-like; trypsin (T)-like, of the Antarctic and temperate 26S isoforms, was performed.

The *D. labrax* 26S proteasome activities did not reveal significant differences in terms of optimal pH values, in opposition to that observed for *C. hamatus* (Figure 3A,B).

In addition, *D. labrax* 26S proteasome showed steeper pH–activity profiles at both sides of the optimum in contrast to *C. hamatus*, possibly ascribed to more susceptibility of the substrate binding sites to the ionic environment changes. As far as the temperature–activity profile is concerned, *D. labrax* holoenzyme displayed the optimum activity of its catalytic subunits in the range 55–60°C, in contrast to the optimal temperatures showed by *C. hamatus* 26S hydrolysing activities, which varied from 37 to 55 °C (Figure 3C,D). These temperatures remained much above those of the cold habitats harboring the psychrophilic organisms as already reported [18,27,28], suggesting that the in vitro assay conditions are often far from those occurring in vivo, as several factors may greatly affect the protein–protein interactions and the enzyme–substrate recognition events. To assess if the dissociation of the 26S complex could contribute to the decreased activities at the extreme pH (5.0 or 10.0) or temperature (10 or 60 °C) values, a Native-PAGE analysis was carried out. As shown in Figure 3E, only the high temperature (60 °C) was able to induce an evident dissociation of 26S proteasome from *C. hamatus* (*D. labrax* 26S complex displayed the same behavior at the extreme pH and temperature values), which could be accountable for the observed reduction in enzyme activities.

**Figure 4:**
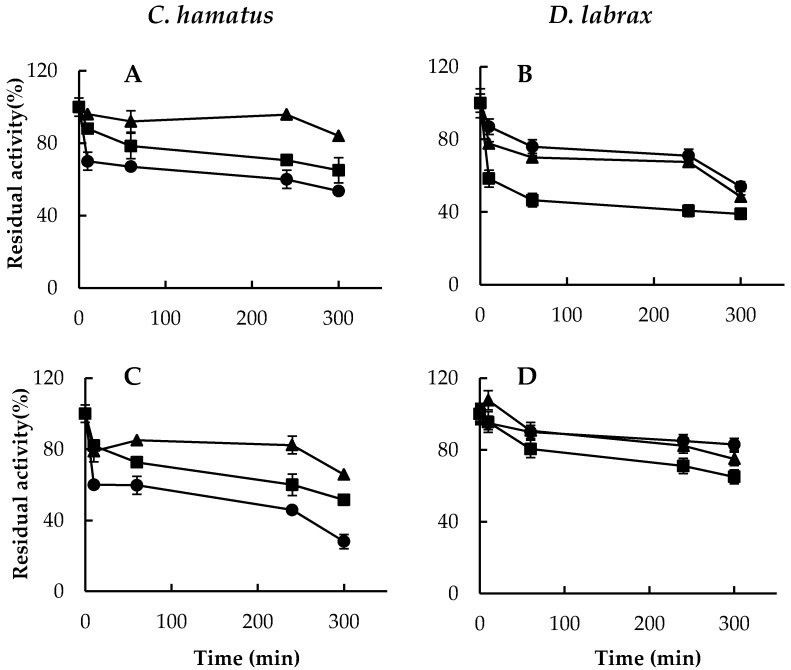
Thermostability of purified 26S proteasomes from *C. hamatus* and *D. labrax*. Thermoresistance of 26S proteasomes at (**A**,**B**) 10 °C; (**C**,**D**) 37 °C. CT-like, PGPH-like and T-like activities are indicated by circle, square and triangle, respectively. Residual activities are expressed as percentage of the corresponding activities at time 0. All experiments were performed in triplicate on three different protein preparations. Data are expressed as means ± standard deviations. Standard deviation values lower than 5% are not shown.

Finally, a greater thermostability at low temperatures (10 °C) was highlighted for all the catalytic subunits of the psychrophilic complex (Figure 4A) compared to those of the temperate counterpart (Figure 4B), which instead, was more stable at increasing temperatures (Figure 4C,D). Interestingly, as already revealed for *T. bernacchii* 26S proteasome [18], the CT-like activity of the cold-adapted enzyme was the least thermostable, indicating a different structural organization of this active site in the cold-adapted complex (Figure 4A–D). However, to determine whether the reduction of 26S proteasome activities was associated to some complex dissociation during the incubation time at the temperatures close to the physiological conditions (10 °C and 37 °C), a Native-PAGE analysis was performed. As shown in Figure S2, pre-incubations of the enzyme complexes at both temperatures even up to 24 h did not induce any disassembling of both 26S isoforms from *D. labrax* and *C. hamatus*, therefore suggesting a strong structural integrity of this important player in proteostasis.

### 2.3. H_2_O_2_ Resistance and Degradation of Oxidized Protein by 26S Proteasomes

**Figure 5:**
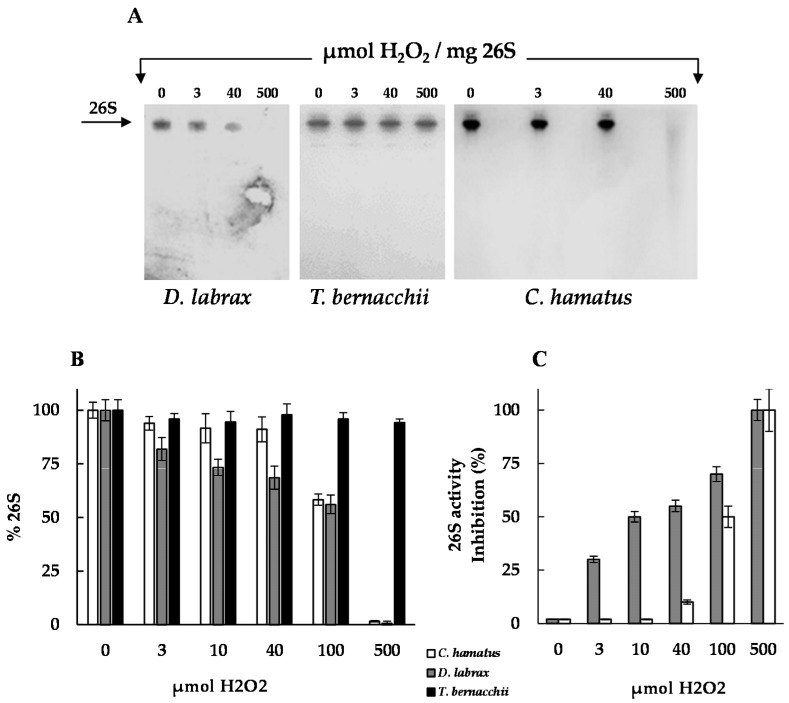
Effects of H_2_O_2_ exposure on *D. labrax* and *C. hamatus* 26S proteasomes. (**A**) Native-PAGE analysis of 26S proteasomes, after incubation with increasing H_2_O_2_ concentrations for 24 h at 37 °C, is shown in comparison with that performed on the *T. bernacchii* 26S proteasome reported in Gogliettino et al. [18]. The arrow indicates the position of 26S proteasome band in each lane. The bands were visualized by Coomassie blue staining. (**B**) Densitometric analysis of Native-PAGE bands were performed by CHEMIDOC XRS and QUANTITY ONE software and expressed as percentage density of the 26S proteasome gel band at all the H_2_O_2_ concentrations tested. The experiments were performed in duplicate on two different protein preparations loading equal amounts of 26S proteasomes. Densitometric data are expressed as means ± standard deviations. (**C**) CT-like 26S proteasome activities after incubation at the indicated H_2_O_2_ concentrations for 24 h. Data were expressed as % of 26S activity inhibition.

In mammalian cells, only the 20S proteasome has been shown to recognize and selectively degrade oxidatively damaged proteins via ubiquitin-independent processes. Accordingly, the ubiquitin-proteasome machinery is much more susceptible than the 20S proteolytic “core” to oxidative stress [10]. We recently suggested a possible involvement of 26S proteasome in the ubiquitin-independent pathways for the degradation of oxidized proteins, in a fish living under cold-induced stress conditions such as *T. bernacchii* [18]. This study raised the question if this “atypical” property of 26S proteasome is the result of cold-adaptation or an intrinsic feature of the piscine isoforms. In an attempt to clarify this point, we evaluated the impact of oxidative treatment on 26S proteasome, exposing equal amounts of the purified Antarctic and temperate holoenzymes to different H_2_O_2_ concentrations, since one of the essential requirements for removal of oxidized proteins is that the enzymes involved must be active under oxidative stress. As shown by Native-PAGE and densitometric analyses (Figure 5A,B), H_2_O_2_-exposure resulted in a decrease of the 26S proteasome amount as revealed by a reduction in Coomassie blue staining intensity of the corresponding bands either in *C. hamatus* then *D. labrax*, although to a different extent. These findings can be ascribed to oxidative modifications of amino acids, which are involved in the Coomassie blue staining.

Specifically, the *C. hamatus* proteasome was shown to be highly stable until 40 µmol of oxidant per mg protein, while oxidative damage, resulting in a decrease of *D. labrax* 26S proteasome band, was already evidenced at 3 µmol of H_2_O_2,_ like the mammalian counterparts [29,30]. These results were opposed to those previously described for *T. bernacchii* 26S proteasome [18], which showed an unexpectedly marked H_2_O_2_ resistance both at structural and activity levels even at 500 µmol per mg of enzyme [18]. Notably, the H_2_O_2_ dose-dependent CT-like activity obtained for all the piscine proteasomes was fully in agreement with the electrophoretic behavior detected in the presence of the oxidant (Figure 5). Indeed, while the *C. hamatus* CT-like activity was only affected starting from 100 µmol of oxidant (50% inhibition), the corresponding *D. labrax* activity diminished at 3 µmol of H_2_O_2_ (30% inhibition) (Figure 5).

**Figure 6:**
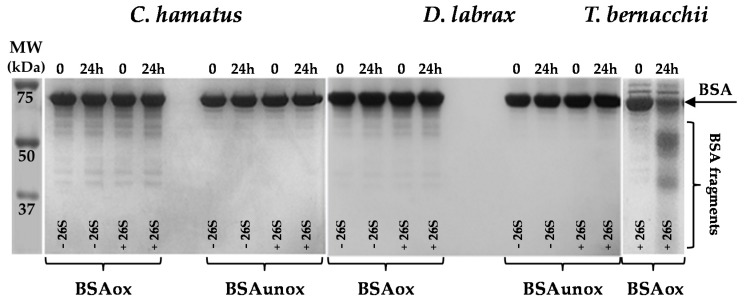
Effects of 26S proteasomes on oxidized or unoxidized bovine serum albumin (BSA) (BSAox or BSAunox, respectively). The arrow indicates the position of BSA band in each lane. Oxidized and unoxidized BSA were incubated at 37 °C for 24 h with or without *C. hamatus* or *D. labrax* 26S proteasome. The reaction mixtures were subjected to SDS-PAGE and the results were compared with those previously reported, showing the degradation pattern of oxidized BSA after incubation with *T. bernacchii* 26S proteasome. The analyses were conducted in triplicate on three different protein preparations.

To better explore the role of the piscine 26S proteasomes in the degradation processes, the enzymes were incubated for 24 h with bovine serum albumin (BSA), previously subjected to oxidative treatment and used as model substrate [18] (Figure 6). The unoxidized BSA was included as negative control and the protein degradation was evaluated by SDS-PAGE analysis. As shown in Figure 6, the intensity of both the oxidized and unoxidized BSA bands, did not decrease and no noticeable breakdown products were detected following the incubation with the icefish or the temperate 26S isoform, which was different to that noticed with *T. bernacchii* 26S proteasome [18], and which was used as positive control.

Based on these results, it can be hypothesized that the adaptation to cold-induced oxidative environments in the Antarctic fish, has caused compensatory adjustments in the 26S proteasome properties, improving its oxidative stress resistance and, in the case of the red-blooded species, antioxidant capacities. The increased levels of oxidative stress in the red-blooded *T. bernacchii*, due to the pro-oxidant action of hemoglobin (Hb) that promotes ROS formation by oxidation of the ferrous iron into the heme prosthetic group, could explain the ‘unexpected’ activity of 26S proteasome against oxidatively damaged proteins [18], in contrast to the isoform from the Hb-less *C. hamatus*, which does not have this capacity. Indeed, many organisms routinely experience wide variation in oxygen availability to their tissues, due to several factors such as environmental high or low oxygen concentrations [16,31]. Recent studies using various animal models (anoxia-tolerant turtles, freeze-tolerant snakes and frogs, estivating snails) explored the adaptation of antioxidant defenses, allowing such organisms to deal with rapid changes in tissue oxygenation with little or no accumulation of damage products [16,31]. For instance, animals that are excellent facultative anaerobes, such as freshwater turtles, during the anoxic–aerobic transition maintain constitutively high antioxidant defenses and associated enzymes, that can readily accommodate the burst of ROS generation. However, although several reports have been focused on the biochemical adaptations that animals have developed to withstand environmental extremes, very little is known about the effects that the life under these harsh conditions have had on the proteostasis processes and protein degradation machineries [16,31].

### 2.4. Cloning and Sequence Analysis

The complementary DNAs (cDNAs) of the proteasomal catalytic subunits of *C. hamatus* were cloned by reverse transcriptase (RT)-polymerase chain reaction (PCR) from whole blood RNAs. The homologous genes of *D. labrax* were retrieved by analysis of the whole genome shotgun sequence [32] (genomic/13489/GCA_000689215.1; seabass_V1.0 GenBank assembly). The corresponding amino acid sequences of each *C. hamatus* and *D. labrax* βchain, which presented the equal number of residues (237 for β1; 199 for β2; 271 for β5), were aligned with the homologues from *T. bernacchii* [18] (Figure S3). The strong conservation of these sequences was evident at first sight, with very high percentages of identity, ranging from 91 to 99%. The β5 proteins were found to be the most conserved (Figure S1C), followed by the β2 and β1 subunits (Figure S1B). All the substitutions were conservative or semi-conservative, excluding few of them in the β5 N- and C-terminal domains (Figure S1C) and in the β1 N-terminal region (Figure S1A), which introduced a change in the amino acid polarity.

### 2.5. Protein Modeling

**Figure 7:**
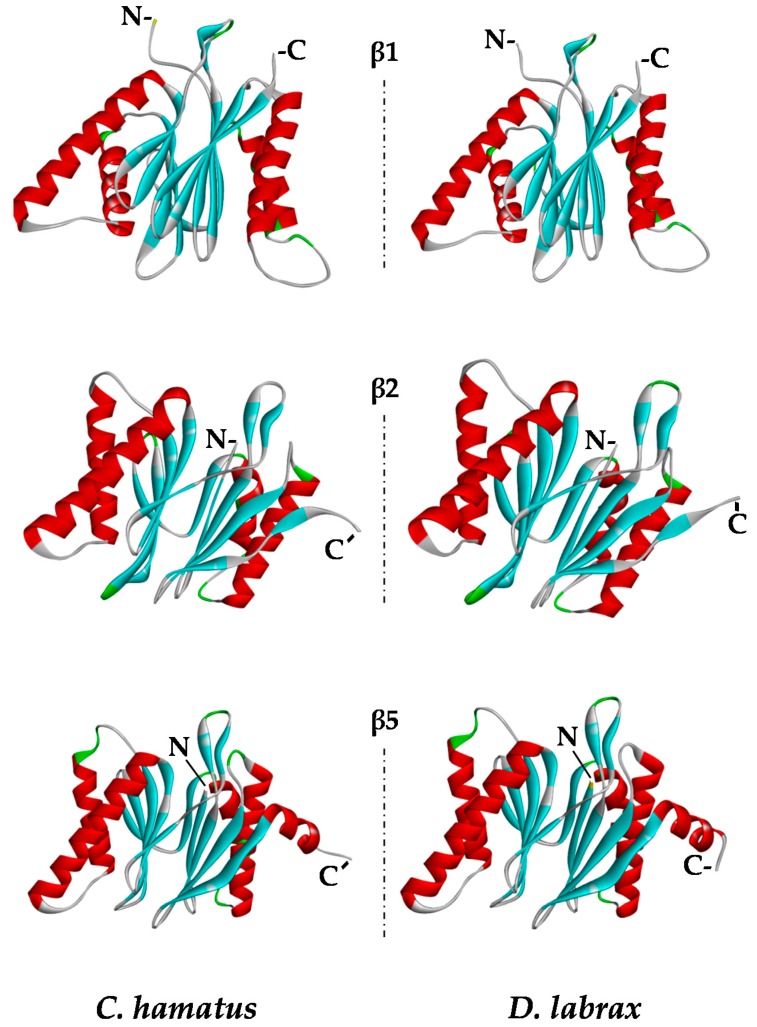
Structural models of β1, β2, and β5 proteasome subunits. α Helix, β-strand and turn structures are indicated with red, cyan and green colors, respectively. N- and C-termini are indicated by N- and C-, respectively. Models were generated as described in the Materials and Methods section. Images were created with Discovery Studio software.

In order to explore the structural features of the catalytic subunits of *C. hamatus* and *D. labrax* proteasomes, we generated the three-dimensional (3D) models of β1, β2, and β5 proteins (Figure 7), comparing them with those previously reported for *T. bernacchii* [18]. The models were built using as templates the 3D structures of the corresponding proteins from *Mus musculus*, which showed high sequence identity (ranging in 70–80%) and sequence coverage (>90%) with the piscine subunits under investigation. The applied modeling approach created high score models via the selection of the best structures in terms of stereochemical and energetical properties. As expected on the basis of the high sequence identity of the β subunits, the 3D structures were very similar in the three piscine organisms. However, to explore the potential alterations in the stabilizing factors, such as intrachain H-bonds and salt-bridges, an in depth comparative examination of the structural model properties was performed (Table 1). The total number of H-bonds observed in the three catalytic subunits of *C. hamatus* and *D. labrax* (483 and 486, respectively) is very similar, differing of only three H-bonds corresponding to about 0.6% of the total. However, the high number of H-bonds makes it very difficult to dissect in detail their geometrical properties, and the very low difference in percentage suggests that intrachain H-bonds in these subunits can similarly contribute to the proteasome stability. On the other hand, the total number of H-bonds observed in the corresponding chains of *T. bernacchii* is 503, which represents an increment of about 5% [18] (Table 1). Therefore, the three subunits in *C. hamatus* and *D. labrax* seem to be similarly stabilized but to a lesser extent than those of *T. bernacchii*, at least concerning the H-bonds.

Looking at the salt bridge interactions, more interesting information was reached. Specifically, a total of 22 and 20 salt bridges was observed in *C. hamatus* and *D. labrax* subunits, respectively, in comparison to the 25 salt bridges present in *T. bernacchii*, which presents an increase of 10%, thus suggesting a higher stability for the three catalytic subunits of this organism. However, to gain more detailed insights into salt bridges, the pairs of amino acids involved in their formation were analyzed and listed in Table 2.

As shown, most of the salt bridges are conserved among the three fish species. Specifically, five salt bridges were well conserved in all β1 chains, whilst a number of further salt bridges were not conserved in *D. labrax* (one), *C. hamatus* (two) and *T. bernacchii* (four), respectively. In β2 chain, a more intricate network of salt bridges was observed, with only five of them conserved among the three species and more different salt bridges which appeared specific for each species. Moreover, in β5 subunit, three out of five salt bridges observed in *D. labrax* and *C. hamatus* were conserved also in *T. bernacchii*, while other salt bridges again were unique for each organism. Overall, the analysis of the intrachain interactions suggests that stability of the single chains may differ among these organisms, possibly with effects on the functional properties. However, without the determination of the whole structure of proteasome of these species, a further hypothesis based on the comparison of putative inter-chain interactions appears too speculative.

These observations support the idea that the extent of variation in the stabilizing interaction networks within the catalytic subunits can be translated into an improvement of the protein architecture, inducing more compact conformations with higher stress resistance of the entire proteasomal complex, as evidenced in the Antarctic fish respect to the temperate counterpart. On the basis of these data, a ‘scale’ of structural stability (26S *T. bernaccchi* > 26S *C. hamatus* > 26S *D. labrax*), which parallels one of the physiological stress conditions, can be proposed.

Future work on reconstitution of Antarctic fish 26S proteasome will be necessary to address how the subunits interact with each other in the protein complex, guaranteeing the stability and functionality of the holoenzyme.

## 3. Materials and Methods

### 3.1. Ethical Procedures

The sample collection and experimental research conducted on the animals utilized in this study were according to the law on activities and environmental protection in Antarctica approved by the Ministry of Foreign Affairs of the Republic of Italy (MAE), to comply with the “Protocol on Environmental Protection to the Antarctic Treaty”, Annex II, Art. 3. All procedures, including euthanasia, were reviewed and approved by MAE and performed in accordance with the European Communities Council Directive of 24 November 1986 (86/609/EEC).

### 3.2. Animal Sampling

Specimens of *C. hamatus* were fished in the vicinity of Mario Zucchelli Station, along the coast of Terra Nova Bay (74′42° S, 164′07° E), Antarctica, during the Italian XXIX expeditions (January–February 2014). Fish were maintained in running seawater at −2 °C to +1 °C until tissue sampling. *D. labrax* specimens were collected at a fish farm, where the water temperature was maintained at 18 °C. The animals were anesthetized with tricaine methanesulphonate (MS222, 300 mg/L) for at least 10 min before being killed by truncation of the spinal cord. Blood was drawn from the caudal vein with heparinized syringes. Blood cells were collected by centrifugation at 3000× *g* for 5 min, washed in 1.7% NaCl, frozen in liquid nitrogen and then stored at −80 °C until use.

### 3.3. 26S Proteasome Preparation

Samples of fresh blood were obtained from the caudal vein with heparinized syringes. *C. hamatus* and *D. labrax* hemolysates were prepared from blood cells, separated from the plasma by centrifugation (1067× *g*, 5 min), and washed twice with cold isotonic solution (10 mM Tris-HCl, pH 7.6, 1.7% NaCl). Lysis of the cells was carried out by incubation in hypotonic solution (25 mM Tris-HCl, pH 7.5) for 30 min on ice. The ‘soluble fraction’ was obtained by centrifugation of the lysate at 9200× *g* for 40 min at 4 °C. The proteasome active fractions, recovered after each purification step, were detected by measuring the CT-like activity using the specific fluorogenic substrate LLVY. The fluorescence intensity owing to 7-amino-4-methylcoumarin (AMC; excitation, 380 nm; emission, 460 nm) was determined using a Jasco FP-8200 spectrofluorometer (Easton, MD, USA). All reagents were purchased from Sigma-Aldrich (Milan, Italy).

#### 3.3.1. *Dicentrarchus labrax*

ATP and glycerol were added to buffers all along the procedure to preserve the interactions between the 20S core and 19S regulatory particles and therefore to maintain 26S proteasome stability [22]. The 26S proteasome purification was performed as previously reported [18] with slightly modifications. The active fractions recovered after the chromatography on Phenyl Sepharose column were dialyzed against 25 mM Tris-HCl, pH 7.5 and loaded onto a Superose 6 PC 3.2/30 column (Pharmacia Biotech, Pittsburgh, PA, USA) connected to a SMART System (Pharmacia Biotech). The elution buffer was 25 mM Tris-HCl, pH 7.5 added with 50 mM NaCl and the flow rate was 0.1 mL·min^−1^. Active fractions were pooled and the purified proteasome was stored in 25 mM Tris-HCl pH 7.5, 1 mM ATP and 10% glycerol.

#### 3.3.2. *Chionodraco hamatus*

The 26S proteasome was purified from the erythrocytes-like cells using the same procedure developed for the *D. labrax* 26S proteasome with slight modifications. Buffers used during the purification steps omitted the use of glycerol and ATP due to remarkable structural stability of *C. hamatus* holoenzyme. The purified proteasome was stored in 25 mM Tris-HCl pH 7.5 and 5% glycerol.

### 3.4. Molecular Mass Determination

Molecular mass of the native 26S proteasome was established by gel filtration chromatography under native conditions on Superose 6 PC 3.2/30 columns (Pharmacia Biotech), connected to a SMART System (Pharmacia Biotech), equilibrated in 25 mM Tris-HCl, pH 7.5 added with 50 mM NaCl, and calibrated with molecular mass standards (26S human proteasome 2100 kDa, 20S human proteasome 700 kDa, apoferritin 443 kDa, porcine Acylpeptide hydrolase 300 kDa, bovine serum albumin 66.5 kDa and chymotrypsin 25 kDa).

### 3.5. 26S Proteasome Peptidase Assay

Enzyme assays were performed by spectroscopic fluorescence as previously reported [18], using the typical substrates for the detection of the three proteasome activities: LLVY for CT-like activity, LRR (*tert*-butyloxycarbonyl-Leu-Arg-Arg-7-amido-4-methylcoumarin; Boston Biochem, (Cambridge, MA, USA) for T-like activity, and LLE (*tert*-butyloxycarbonyl-Leu-Leu-Glu-7-amido-4-methylcoumarin; Sigma-Aldrich) for PGPH-like activity. All the enzymatic activities were expressed in arbitrary units (assuming ε = 1 mM^−1^·cm^−1^). All the experiments were carried out in triplicate on three different protein preparations. The reaction mixture (0.8 mL), containing the appropriate amount of enzyme in 50 mM Tris-HCl buffer at optimal pH and temperature was pre-incubated for 5 min. Then, the specific substrate was added and the release of product was measured.

### 3.6. pH and Temperature Effects on Proteasome Activities

The assays were performed as previously described [18]. Effect of pH ranging from 5.0 to 10.0 was analyzed at 37 °C, whilst the effect of temperatures ranging from 10 to 60 °C was investigated at pH 7.5. Blanks that contained no enzyme were used to subtract a blank rate at each pH and temperature value. Thermal stability was determined by measuring activities after incubation of the enzyme at 10 °C and 37 °C.

### 3.7. Gel Electrophoresis

SDS-PAGE (12%) was carried out according to the procedure described before [18]. Native-PAGE (4.6%) was performed according to the method described by Holzl et al. [33]. Proteasome activity was detected by in-gel peptidase activity performed as previously reported [23] with some modifications. Specifically, the gel was immersed in 50 mM Tris-HCl, pH 8.0 and 100 µM LLVY in the presence or absence of 0.02% of SDS, at 37 °C for 1 h. The fluorescence was detected 30 min after exposure to the fluorogenic peptide.

Native-PAGE analyses of 26S proteasomes pre-incubated at pH 5.0, pH 10.0, 10 °C or 60 °C under the peptidase assay conditions described above, were carried out as describe in Holzl et al. [33]. In addition, Native-PAGE of 26S proteasomes after prolonged incubation (up to 24 h) at 10 °C or 37 °C, was performed in the same experimental conditions [33].

### 3.8. Western Blot Analysis

Samples were run on Native-PAGE (4.6%) and then electroblotted on to polyvinylidene difluoride (PVDF) membranes (Immobilon^TM^, Millipore, Billerica, MA, USA). Membranes were next incubated with the piscine anti-β1/β5 (LSC111925 rabbit IgG, 1:1000, LifeSpan BioSciences, Seattle, WA, USA) and piscine anti-Rpt1 (LS-C290473 rabbit IgG, 1:1000, LifeSpan BioSciences) primary antibodies (1 h at room temperature) and then with the horseradish peroxidase (HRP)-conjugated secondary antibodies (1 h at room temperature). Immune complexes formed were visualized by enhanced chemiluminescence and autoradiography (Amersham Biosciences, Little Chalfont, UK). Protein expression data were quantified with Quantity One Software (Bio-Rad Laboratories, Hercules, CA, USA).

### 3.9. Oxidant Resistance of Proteasome and Degradation of Oxidized Bovine Serum Albumin by Proteasome

The oxidant resistance and the degradation of oxidized BSA by 26S proteasomes were tested as previously reported by the authors of [18].

### 3.10. Cloning

Total RNA from whole blood of *C. hamatus* and from liver of *D. labrax* was isolated according to the RNeasy Plus Universal Mini Kit (Qiagen, Hilden, Germany) protocol. RNA concentration was determined with a Qubit Fluorometer (Invitrogen, Carlsbad, CA, USA). RNA was then reverse transcribed with the SuperScript VILO MasterMix (Invitrogen). The cDNAs of the catalytic proteasome subunits were PCR amplified with primers already designed on the homologues sequences from the notothenioids *T. bernacchii* [18] and *Notothenia coriiceps*, and on the Basic Local Alignment Search Tool (BLAST) analysis of several SRA libraries from *Notothenioidei* (SRX088548, SRX089044-9, SRX373094-100, SRX305406, SRX306432, SRX306459, SRX306462-4) and of the *D. labrax* whole genome shotgun sequence (genomic/13489/GCA_000689215.1; seabass_V1.0 GenBank assembly) [32].

The oligonucleotides are listed in Table 3. The amplifications were performed as follows: 94 °C for 2 min, 40 cycles of 94 °C (30 s), 58–62 °C (30 s), and 72 °C (1 min), and a final extension at 72 °C for 10 min. The PCR amplicons were analyzed on 1% agarose gel, purified with the Zymoclean Gel DNA Recovery Kit (Zymo Research, Irvine, CA, USA), and cloned into the StrataClone PCR Cloning kit (Stratagene, San Diego, CA, USA).

### 3.11. Sequence Analysis

The cDNAs were analyzed by automated sequencing at Eurofins Genomics GmbH (Ebersberg, Germany). The sequences were edited and analyzed with the CLC Main Workbench 7.7 program (CLC bio, 2016) and deposited in the GenBank database under the accession numbers KY306449 (Cham_beta1), KY306450 (Cham_beta2), KY306451 (Cham_beta5) for *C. hamatus*, and in the Third Party Annotation Section of the DDBJ/ENA/GenBank databases, under the accession numbers BK009988 (Dlab_beta1), BK009989 (Dlab_beta2), and BK009990 (Dlab_beta5) for *D. labrax*.

### 3.12. Molecular Modeling

Molecular modeling techniques were used to generate 3D models of the *C. hamatus* and *D. labrax* catalytic subunits, accordingly to procedure previously applied [18]. Briefly, template structures were selected from the Protein Data Bank (PDB) [34] on the basis of the best sequence similarity. The mouse proteasome structure (PDB ID: 3UNB) were selected to this scope. Very high sequence similarity exists between each fish subunit sequence and the corresponding mouse subunit (ranging between 70% and 80% sequence identity) so that sequence alignment does not require gap insertion. For β5 subunit, the N-terminal segment of 68 amino acids was not included in the model, due to the lack of this region in the template X-ray structure. For each subunit, 10 models were created by Modeller 9.12 [35]. The best model for each subunit was selected by checking stereochemical and energetic properties, by means of PROCHECK [36] and PROSA-web [37] software, respectively. Final models were analyzed in detail looking at the potential H-bonds and salt bridges by means of HBplus [38] and an original software developed at CNR-ISA Bioinformatics and Computational Biology Lab.

## 4. Conclusions

The generation of ROS is an inevitable aspect of aerobic life and of the cellular metabolism. In the Antarctic fish, exposure to extremely oxidant environments due to the cold water temperatures [39,40,41] results in higher oxidative stress levels compared to the temperate habitats [14,42,43,44]. This causes a progressive protein impairment in cells [39], which are thus equipped with several defense mechanisms needed to counteract the oxidative damage and accumulation of toxic protein aggregates [14,45,46,47,48]. In mammals, the 20S proteasome is the major proteolytic machinery deputed to the degradation of the oxidatively modified proteins via ATP- and ubiquitin-independent pathways [2,5,8,9]. In the present study, we have provided new insights into the role played by the cold-adapted 26S proteasome in the oxidative stress response, assuming its direct involvement in the antioxidant processes, being able to efficiently degrade oxidized proteins in ATP- and ubiquitin-independent manner and/or markedly resist to oxidants. This is in contrast to that which has been reported for mammalian 26S isoforms, which contributes to protein degradation machinery by ATP- and ubiquitin-dependent pathway and has shown to be very sensitive to inactivation by oxidants [2,5,8,9]. Indeed, it has been reported that the degradation of oxidatively damaged proteins does neither require the presence of ATP nor the polyubiquitination of the substrate [3,5]. In fact, the 26S proteasome disassembles and the ubiquitinating system becomes already deactivated in H_2_O_2_ concentrations that are about one magnitude lower than the concentrations needed to inactivate the uncapped 20S proteasome [3,5]. Therefore, 20S has been recognized as the key factor of the degradation of oxidatively damaged proteins, due to a substrate recognition mechanism possibly mediated by the exposure of hydrophobic structures that is normally buried inside of a natively folded protein [3]. Hence, our findings uncover an until now, unexplored degradation mechanism of oxidized proteins involving the 26S proteasome, gaining a new perspective on the impact that the cold-adaptation and the evolutionary pressure to oxidant environments that may have taken place on the structural and functional features of the main cytosolic proteolytic system.

## Figures and Tables

**Table 1:** Intrachain H bonds and salt bridges in *C. hamatus* and *D. labrax* subunits in comparison with those of *T. bernacchii*.

	β1	β2	β5
**Intrachain H Bonds**			
***C. hamatus***	164	151	168
***D. labrax***	169	150	167
***T. bernacchii***	169	158	176
**Intrachain Salt Bridges**			
***C. hamatus***	7	10	5
***D. labrax***	6	9	5
***T. bernacchii***	9	10	6

**Table 2:** Pairs of amino acids involved in salt bridges.

Chain	*C. hamatus*	*D. labrax*	*T. bernacchii*
**β1**	**Glu184||Arg211**	**Glu184||Arg211**	**Glu184||Arg211**
	**Glu18||Lys118**	**Glu18||Lys118**	**Glu18||Lys118**
	**Asp114||Arg132**	**Asp114||Arg132**	**Asp114||Arg132**
	**Glu31||His36**	**Glu31||His36**	**Glu31||His36**
	**Asp191||Arg28**	**Asp191||Arg28**	**Asp191||Arg38**
	Glu205||Lys45	-	-
	Asp213||Arg211	-	-
	-	Asp114||His116	-
	-	-	Glu205||Arg194
	-	-	Asp150||Lys156
	-	-	Asp191||Arg28
	-	-	Asp133||Lys136
**β2**	**Asp184||His189**	**Asp184||His189**	**Asp184||His189**
	Asp90||Lys86	Asp90||Lys86	-
	**Glu40||Lys37**	**Glu40||Lys37**	**Glu40||Lys37**
	**Asp18||Lys34**	**Asp18||Lys34**	**Asp18||Lys34**
	Glu74||Lys68	Glu74||Lys68	-
	**Asp33||Arg181**	**Asp33||Arg181**	**Asp33||Arg181**
	**Asp184||Arg153**	**Asp184||Arg153**	**Asp184||Arg153**
	Asp190||Arg181	-	-
	Glu109||Lys41	-	Glu109||Lys41
	ASP 52||HIS 99	-	-
	-	Glu166||Arg170	-
	-	Asp11||Arg153	-
	-	-	Glu186||His189
	-	-	Asp31||Lys29
	-	-	Glu58||Lys62
	-	-	Glu109||Lys185
**β5**	**Asp17||Lys33**	**Asp17||Lys33**	**Asp17||Lys33**
	**Glu67||Arg64**	**Glu67||Arg64**	**Glu67||Arg64**
	**Glu36||Arg186**	**Glu36||Arg186**	**Glu36||Arg186**
	Glu117||Lys91	-	Glu117||Lys91
	Glu182||Arg183	-	-
	-	Asp124||Lys7	-
	-	Glu190||Arg157	-
	-	-	Asp105||Arg107
	-	-	Glu154||Arg157

Conserved pair amino acids involved in salt bridges are highlighted in bold.

**Table 3:** Primers designed for the amplifications of the *C. hamatus* and *D. labrax* catalytic proteasome subunits cDNAs.

Primer	Sequence	Tm (°C)
**Chbetalfor**	5′-CCATATTGCAGTGATACAGCGAG-3′	60.5
**Chbetalrev**	5′-CTCAGTCCTTCCTCAGGGG-3′	60.1
**Chbeta2for**	5′-GTCGGGATACAGGGACCG-3′	60.8
**Chbeta2rev**	5′-AGCGGTCACTTGGCGC-3′	62.8
**Chbeta5for**	5′-GGGAGTTTCAAAGATGGCTCT-3′	59.2
**Chbeta5rev**	5′-TTGTACTGCTGGTGCAGCAT-3′	61.7
**Dlbetalfor**	5′-ATGATTTCTGCTCATGGTTTCGG-3′	60.6
**Dlbetalrev**	5′-CCCTCCTCAGTCCTTCCTC-3′	59.8
**Dlbeta2for**	5′-GCACCATGGAGTATTTAATCGGG-3′	60.4
**Dlbeta2rev**	5′-GTGAGCGGTCACTTGGC-3′	60.4
**Dlbeta5for**	5′-ATGGCTCTTGCTAGTGTGTTG-3′	59.8
**Dlbeta5rev**	5′-CATGACTATGCCTGATCTTTGTACTG-3′	60.3

## Supplementary Materials

Supplementary materials can be found at www.mdpi.com/1422-0067/18/8/1605/s1.
