# Supplementary material for: Unusual Antioxidant Properties of 26S Proteasome Isolated from Cold-Adapted Organisms
Source: Int J Mol Sci. 2017 Jul 25;18(8):1605. doi: 10.3390/ijms18081605 (PMC5577997; doi:10.3390/ijms18081605)
Supplement: Supplementary file 1 [file ijms-18-01605-s001.pdf]

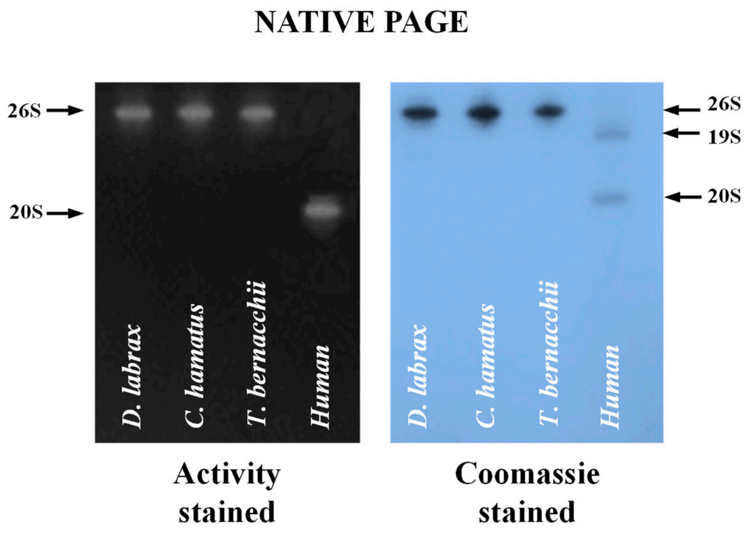

Figure S1. Native-PAGE analysis of purified 26S proteasomes from *D. labrax* and *C. hamatus* in comparison to those from *T. bernacchii* and human. In-gel CT-like activity (β5 subunit) of 26S proteasomes was detected by using the fluorogenic substrate LLVY in the presence of 0.02% SDS. The Coomassie blue stained Native-PAGE was also shown. 26S proteasome from *T. bernacchii* and human were used as positive controls. The main proteasome species are indicated: doubly capped 26S (19S-20S-19S); 19S (regulatory complex) and 20S (catalytic core), free particles.

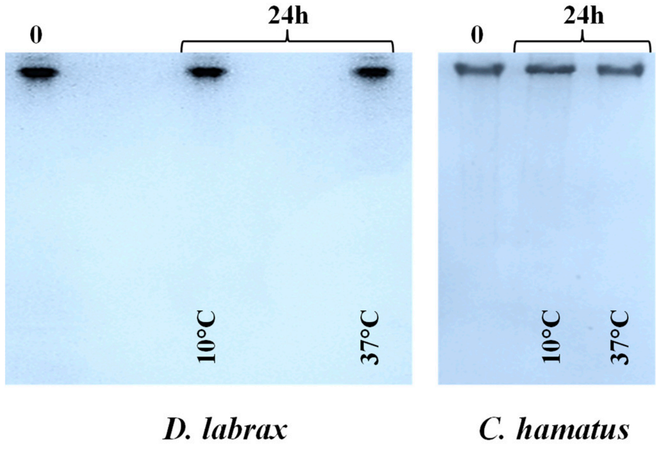

Figure S2. Effects of temperature on *D. labrax* and *C. hamat*us 26S proteasomes. Native-PAGE analyses of purified 26S proteasomes after incubation at 10 °C and 37 °C for 24 h are shown. The bands were visualized by Coomassie blue staining. The experiments were performed in duplicate on two different protein preparations loading equal amounts of 26S proteasomes.

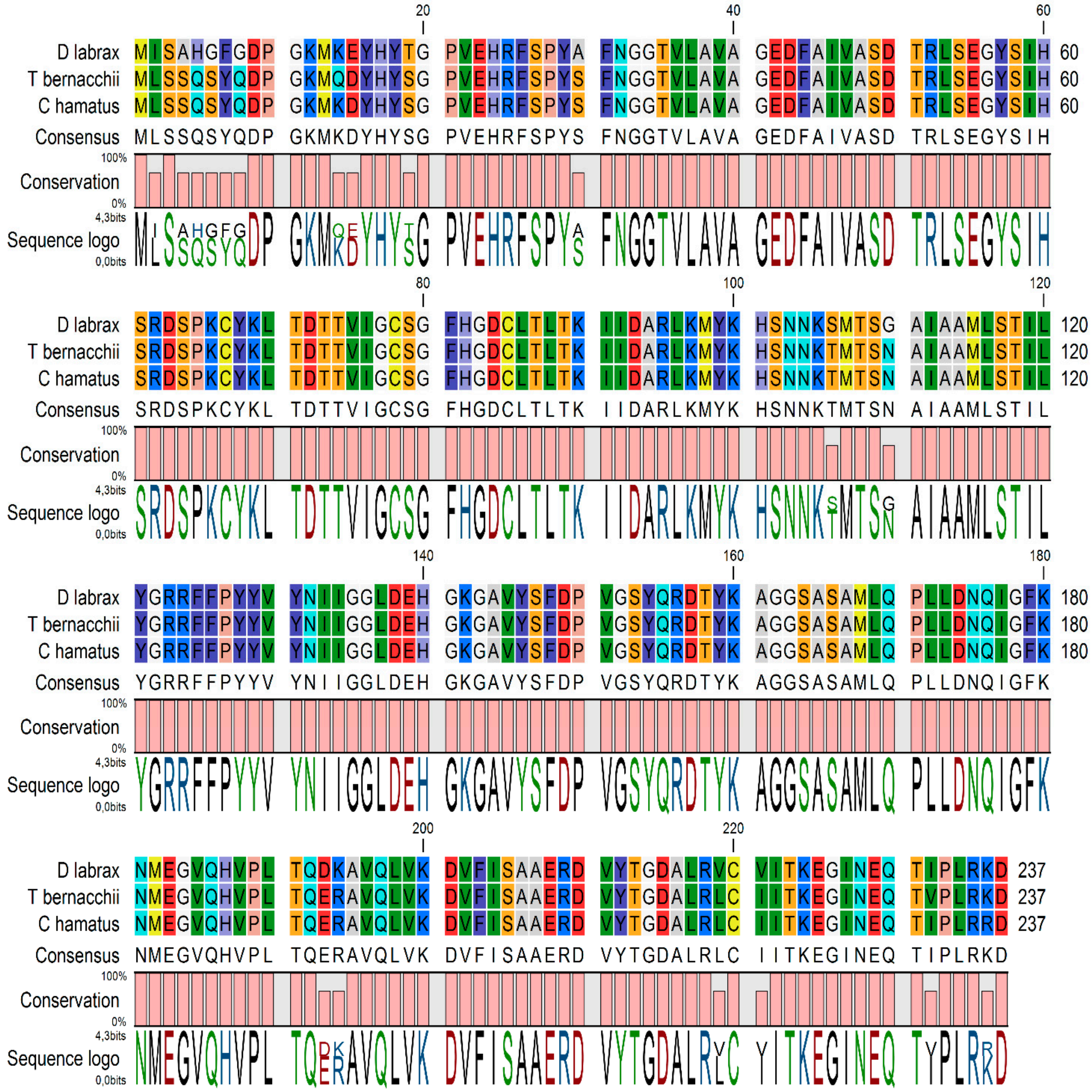
D labrax MISAHGFGDP GKMKEYHYTG PVEHRFSPYA FNGGTVLAVA GEDFAIVASD TRLSEGYSIH 60
T bernacchii MLSSQSYQDP GKMQDYHYSG PVEHRFSPYS FNGGTVLAVA GEDFAIVASD TRLSEGYSIH 60
C hamatus MLSSQSYQDP GKMKDYHYSG PVEHRFSPYS FNGGTVLAVA GEDFAIVASD TRLSEGYSIH 60
Consensus MLSSQSYQDP GKMKDYHYSG PVEHRFSPYS FNGGTVLAVA GEDFAIVASD TRLSEGYSIH
Conservation
Sequence logo
D labrax SRDSPKCYKL TDTTVIGCSG FHGDCLTLTK IIDARLKMYK HSNNKSMTSG AIAAMLSTIL 120
T bernacchii SRDSPKCYKL TDTTVIGCSG FHGDCLTLTK IIDARLKMYK HSNNKTMTSN AIAAMLSTIL 120
C hamatus SRDSPKCYKL TDTTVIGCSG FHGDCLTLTK IIDARLKMYK HSNNKTMTSN AIAAMLSTIL 120
Consensus SRDSPKCYKL TDTTVIGCSG FHGDCLTLTK IIDARLKMYK HSNNKTMTSN AIAAMLSTIL
Conservation
Sequence logo
D labrax YGRRFFPYYV YNIIGGLDEH GKGAVYSFDP VGSYQRDTYK AGGSASAMLQ PLLDNQIGFK 180
T bernacchii YGRRFFPYYV YNIIGGLDEH GKGAVYSFDP VGSYQRDTYK AGGSASAMLQ PLLDNQIGFK 180
C hamatus YGRRFFPYYV YNIIGGLDEH GKGAVYSFDP VGSYQRDTYK AGGSASAMLQ PLLDNQIGFK 180
Consensus YGRRFFPYYV YNIIGGLDEH GKGAVYSFDP VGSYQRDTYK AGGSASAMLQ PLLDNQIGFK
Conservation
Sequence logo
D labrax NMEGVQHVPL TQDKAVQLVK DVFISAAERD VYTGDALRVC VITKEGINEQ TIPLRKD 237
T bernacchii NMEGVQHVPL TQERAVQLVK DVFISAAERD VYTGDALRLC IITKEGINEQ TVPLRKD 237
C hamatus NMEGVQHVPL TQERAVQLVK DVFISAAERD VYTGDALRLC IITKEGINEQ TIPLRRD 237
Consensus NMEGVQHVPL TQERAVQLVK DVFISAAERD VYTGDALRLC IITKEGINEQ TIPLRKD
Conservation
Sequence logo

A

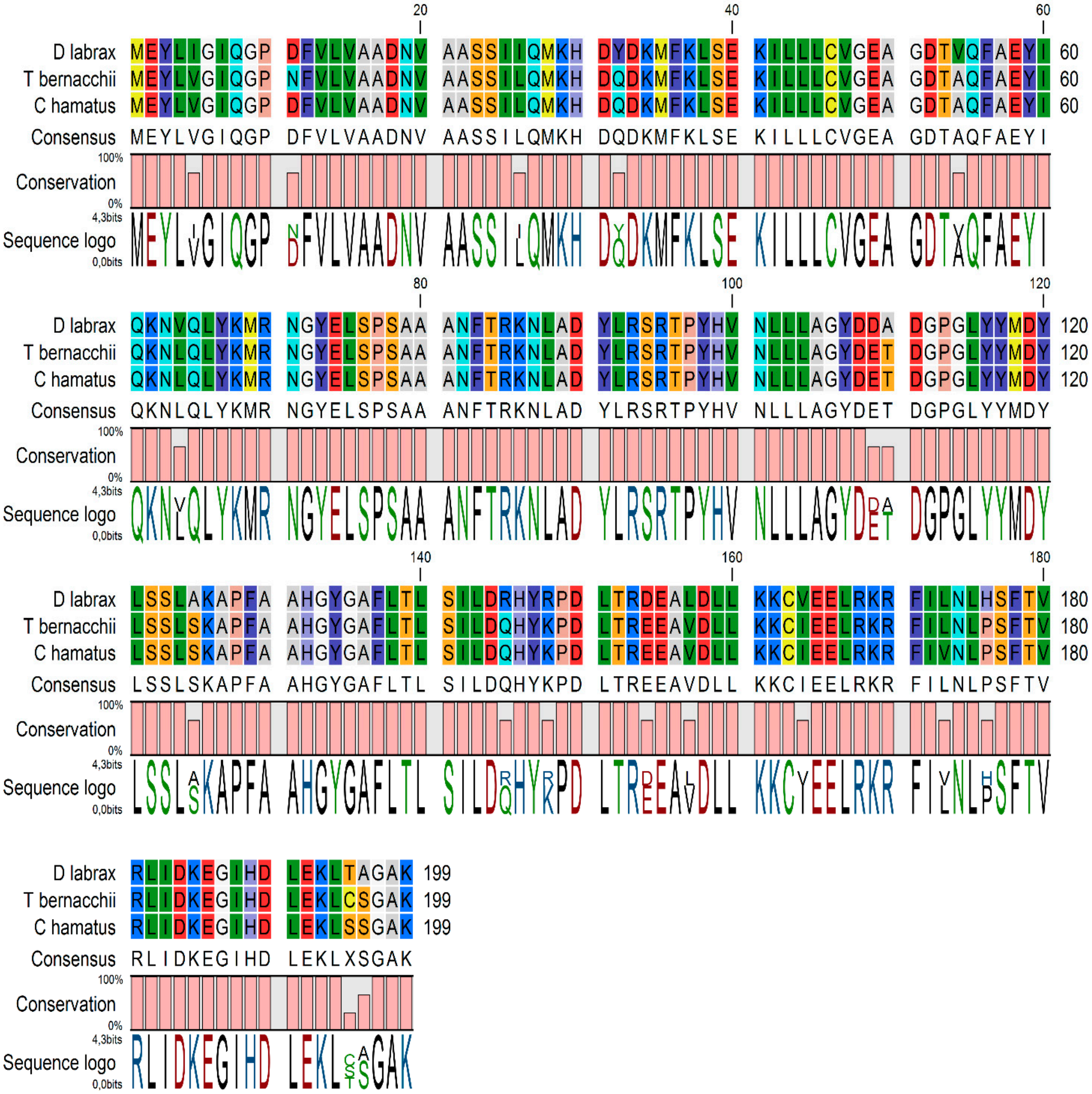
20 40 60
D labrax MEYLIGIQGP DFVLVAADNV AASSIIQMKH DYDKMFKLSE KILLLCVGEA GDTVQFAEYI 60
T bernacchii MEYLVGIQGP NFVLVAADNV AASSILQMKH DQDKMFKLSE KILLLCVGEA GDTAQFAEYI 60
C hamatus MEYLVGIQGP DFVLVAADNV AASSILQMKH DQDKMFKLSE KILLLCVGEA GDTAQFAEYI 60
Consensus MEYLVGIQGP DFVLVAADNV AASSILQMKH DQDKMFKLSE KILLLCVGEA GDTAQFAEYI
100%
Conservation
0%
4,3bits
Sequence logo
0,0bits
80 100 120
D labrax QKNVQLYKMR NGYELSPSAA ANFTRKNLAD YLRSRTPYHV NLLLAGYDDA DGPGLYYMDY 120
T bernacchii QKNLQLYKMR NGYELSPSAA ANFTRKNLAD YLRSRTPYHV NLLLAGYDET DGPGLYYMDY 120
C hamatus QKNLQLYKMR NGYELSPSAA ANFTRKNLAD YLRSRTPYHV NLLLAGYDET DGPGLYYMDY 120
Consensus QKNLQLYKMR NGYELSPSAA ANFTRKNLAD YLRSRTPYHV NLLLAGYDET DGPGLYYMDY
140 160 180
D labrax LSSLAKAPFA AHGYGAFLTL SILDRHYRPD LTRDEALDLL KKCVEELRKR FILNLHSFTV 180
T bernacchii LSSLSKAPFA AHGYGAFLTL SILDQHYKPD LTREEAVDLL KKCIEELRKR FILNLPSFTV 180
C hamatus LSSLSKAPFA AHGYGAFLTL SILDQHYKPD LTREEAVDLL KKCIEELRKR FIVNLPSFTV 180
Consensus LSSLSKAPFA AHGYGAFLTL SILDQHYKPD LTREEAVDLL KKCIEELRKR FILNLPSFTV
D labrax RLIDKEGIHD LEKLTAGAK 199
T bernacchii RLIDKEGIHD LEKLCSGAK 199
C hamatus RLIDKEGIHD LEKLSSGAK 199
Consensus RLIDKEGIHD LEKLXSGAK

B

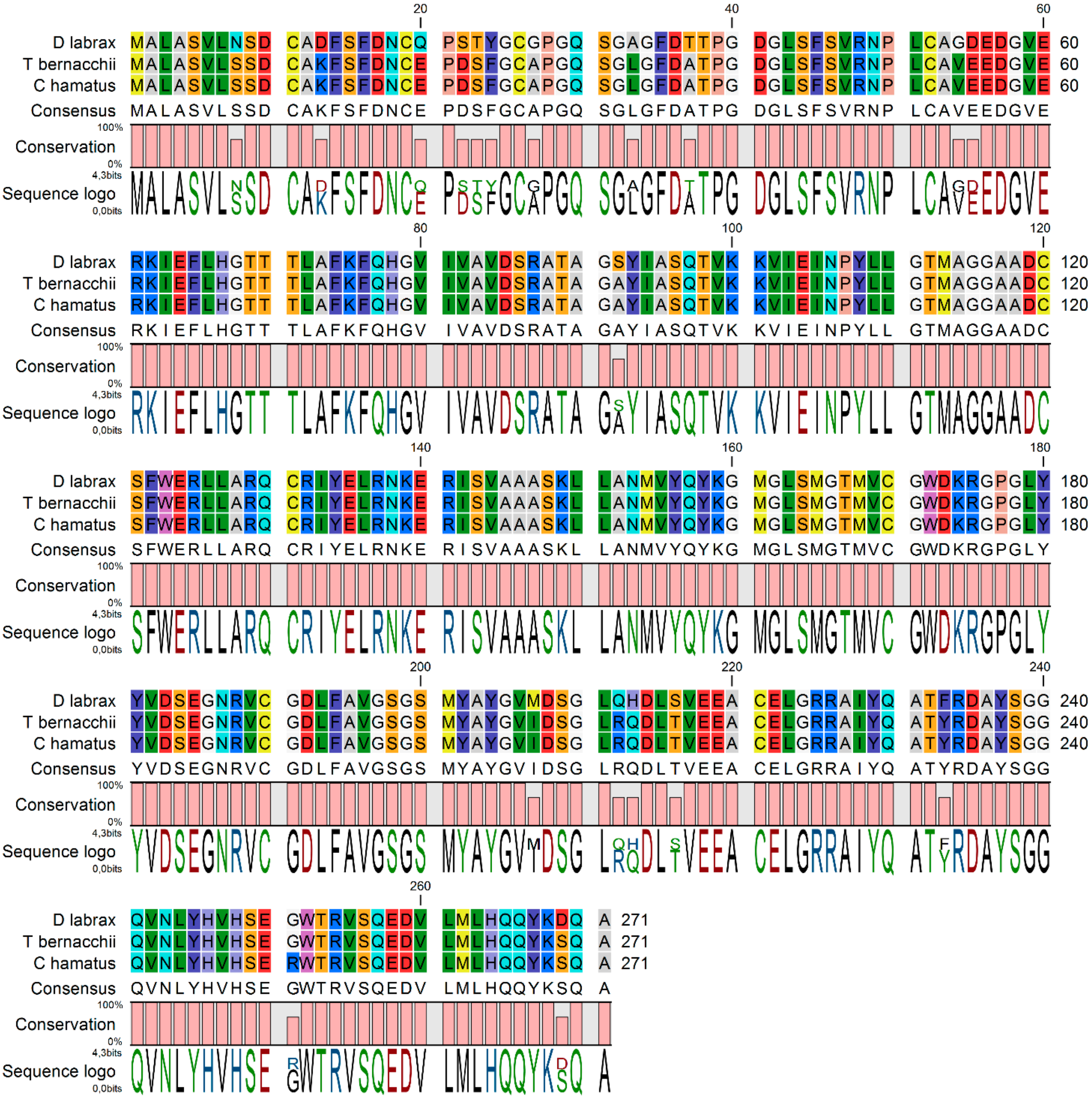

C

Figure S3. MUSCLE alignments of the amino acid sequences of β1, β2, and β5 proteasome catalytic subunits. Alignments of the amino acid translations of *C. hamatus* and *D. labrax* proteasome catalytic β subunits cDNAs with the homologues sequences from *T. bernacchii*. **A**, β1 subunits; **B**, β2 subunits; **C**, β5 subunits. The amino acids are indicated observing the IUPAC codes, where the residues with negative polarity are in shades of red, and those with positive polarity are in blue shades. The consensus sequence, the conservation histogram and the sequence logo are shown at the bottom of the alignments.

Table S1. Purification steps of 26S proteasome from *D. labrax* erythrocytes.

Purification step	Total activity (U)	Total protein (mg)	Specific activity (U/mg)	U/mL blood	Purification fold	Yield (%)
Extract	5.16×10^5	69.00	7.47×10^3	1.84×10^5	1	100
DEAE	2.27×10^5	24.00	9.45×10^3	-	1.3	44
Phenyl Sepharose	6.50×10^4	0.90	7.22×10^4	-	9.7	12
Superose 6	6.50×10^3	0.08	8.10×10^4	-	10.8	1.2

The proteasome active fractions, recovered after each purification step, were detected by measuring the chymotrypsin (CT)-like activity using the specific fluorogenic substrate N-succinyl-Leu-Leu-Val-Tyr-7-amido-4-methylcoumarin and expressed in arbitrary unit.

Table S2. Purification steps of 26S proteasome from *C. hamatus* erythrocytes-like cells.

Purification step	Total activity (U)	Total protein (mg)	Specific activity (U/mg)	U/mL blood	Purification fold	Yield (%)
Extract	1.52×10^6	638.0	2.38×10^3	3.80×10^5	1	100
DEAE	2.89×10^5	68.0	4.25×10^3	-	1.80	19
Phenyl Sepharose	3.59×10^4	10.0	3.6×10^3	-	1.70	2.4
Superose 6	2.69×10^4	3.0	9.0×10^3	-	3.80	1.8

The proteasome active fractions, recovered after each purification step, were detected by measuring the chymotrypsin (CT)-like activity using the specific fluorogenic substrate N-succinyl-Leu-Leu-Val-Tyr-7-amido-4-methylcoumarin and expressed in arbitrary unit.
